# MiR-451a suppressing BAP31 can inhibit proliferation and increase apoptosis through inducing ER stress in colorectal cancer

**DOI:** 10.1038/s41419-019-1403-x

**Published:** 2019-02-15

**Authors:** Ke Xu, Bin Han, Yang Bai, Xiu-Ying Ma, Zhen-Ni Ji, Yao Xiong, Shi-Kun Miao, Yuan-Yuan Zhang, Li-Ming Zhou

**Affiliations:** 1 Department of Pharmacology, West China School of Basic Medical Sciences & Forensic Medicine, Sichuan University, Chengdu, Sichuan 610041 China; 2 985 Science and Technology Platform for Innovative Drugs, Sichuan University, Chengdu, Sichuan 610041 China; 3 State Key Laboratory of Biotherapy and Cancer Center, West China Hospital, Sichuan University, and Collaborative Innovation Center of Biotherapy, Chengdu, Sichuan 610041 China

## Abstract

The global morbidity and mortality of colorectal cancer (CRC) are ranked the third among gastrointestinal tumors in the world. MiR-451a is associated with several types of cancer, including CRC. However, the roles and mechanisms of miR-451a in CRC have not been elucidated. BAP31 is a predicted target gene of miR-451a in our suppression subtractive hybridization library. Its relationship with miR-451a and function in CRC are unclear. We hypothesized that miR-451a could induce apoptosis through suppressing BAP31 in CRC. Immunohistochemistry and real-time PCR were used to measure BAP31 expressions in CRC tissues and pericarcinous tissues from 57 CRC patients and CRC cell lines. Dual-luciferase reporter assay was used to detect the binding of miR-451a to BAP31. The expression of BAP31 protein in CRC tissues was significantly higher than that in pericarcinous tissues, which was correlated with distant metastasis and advanced clinical stages of CRC patients. The expression of BAP31 was higher in HCT116, HT29, SW620, and DLD cells than that in the normal colonic epithelial cell line NCM460. The expression of BAP31 was absolutely down-regulated when over-expressing miR-451a in HCT116 and SW620 cells compared with control cells. Mir-451a inhibited the expression of BAP31 by binding to its 5’-UTR. Over-expressing miR-451a or silencing BAP31 suppressed the proliferation and apoptosis of CRC cells by increasing the expressions of endoplasmic reticulum stress (ERS)-associated proteins, including GRP78/BIP, BAX, and PERK/elF2α/ATF4/CHOP, which resulted in increased ERS, cytoplasmic calcium ion flowing, and apoptosis of CRC cells. These changes resulting from over-expressing miR-451a were reversed by over-expressing BAP31 with mutated miR-451a-binding sites. Over-expressing miR-451a or silencing BAP31 inhibited tumor growth by inducing ERS. The present study demonstrated that miR-451a can inhibit proliferation and increase apoptosis through inducing ERS by binding to the 5’-UTR of BAP31 in CRC.

## Introduction

Colorectal cancer (CRC) is the third most common malignant gastrointestinal tumor in the world. Its mortality has increased from 694,000 in 2012 to 774,000 in 2015, with the increased death ratio being 11.53%^1^. With the improvement of people’s living standards, the incidence and the mortality of CRC in China were both increased to the fifth among all cancers in 2011^2^. The current treatments for CRC include resection, radiotherapy, and chemotherapy. Chemotherapy can be used for patients at different clinical stages, but is not recommended for patients with poor general or organ functions. The recommended initiation of chemotherapy is within 8 weeks after surgery, and the time limit for chemotherapy should be not more than 6 months^3^. Although the response rate to systemic chemotherapy is less than 50%, drug resistance develops in nearly all patients^4,5^. So, there is an urgent need to explore new therapeutic targets for CRC to improve clinical efficacy.

Micro RNA (microRNA; miRNA), consisting of about 21–23 nucleotides, is a eukaryotic ubiquitous endogenous small RNA. MiRNA gene is a highly conserved gene family, which is involved in multiple biological processes such as proliferation, apoptosis, and senescence. MicroRNA-451a (miR-451a) has been reported to be significantly down-regulated in chronic myeloid leukemia, glioma, non-small cell lung cancer, gastric cancer, and breast cancer. It can inhibit the proliferation, invasion, and metastasis of tumor cells, and increase the apoptosis and improve the therapeutic effects of radiotherapy and chemotherapy^6–14^. However, its role and target genes in CRC have not been elucidated, yet. Our previous report has demonstrated that the expression of miR-451a in CRC tissues was significantly down-regulated compared to pericarcinous tissues of 68 CRC patients. The expression of miR-451a was decreased in HCT116, SW620, HT29, SW480, and DLD cells compared with the normal colonic epithelial cell NCM460^15^. Therefore, we believed that miR-451a, as a tumor suppressor, plays an important role in the carcinogenesis of CRC. We also predicted seven potential target genes of miR-451a in CRC by our suppression subtractive hybridization method^15^.

BAP31, one of our predicted target genes of miR-451a, located in the endoplasmic reticulum, is an important molecular chaperone protein^16,17^. As a carrier protein, BAP31 plays an important role in apoptosis^18–20^. The expression of BAP31 protein was dramatically up-regulated in human malignant melanoma tumor tissues and human primary hepatocellular carcinoma when compared with normal tissues^21,22^. However, the roles of BAP31 in CRC remain unclear. Whether or not it is a target of miR-451a remains undetermined.

In the present study, we aim to investigate the effects and mechanisms of BAP31 in CRC in vivo and in vitro, and how miR-451a regulates the expression of BAP31.

## Results

### Elevated BAP31 expression in CRC tissues was correlated with advanced clinical stage and distant metastasis

**Fig. 1:**
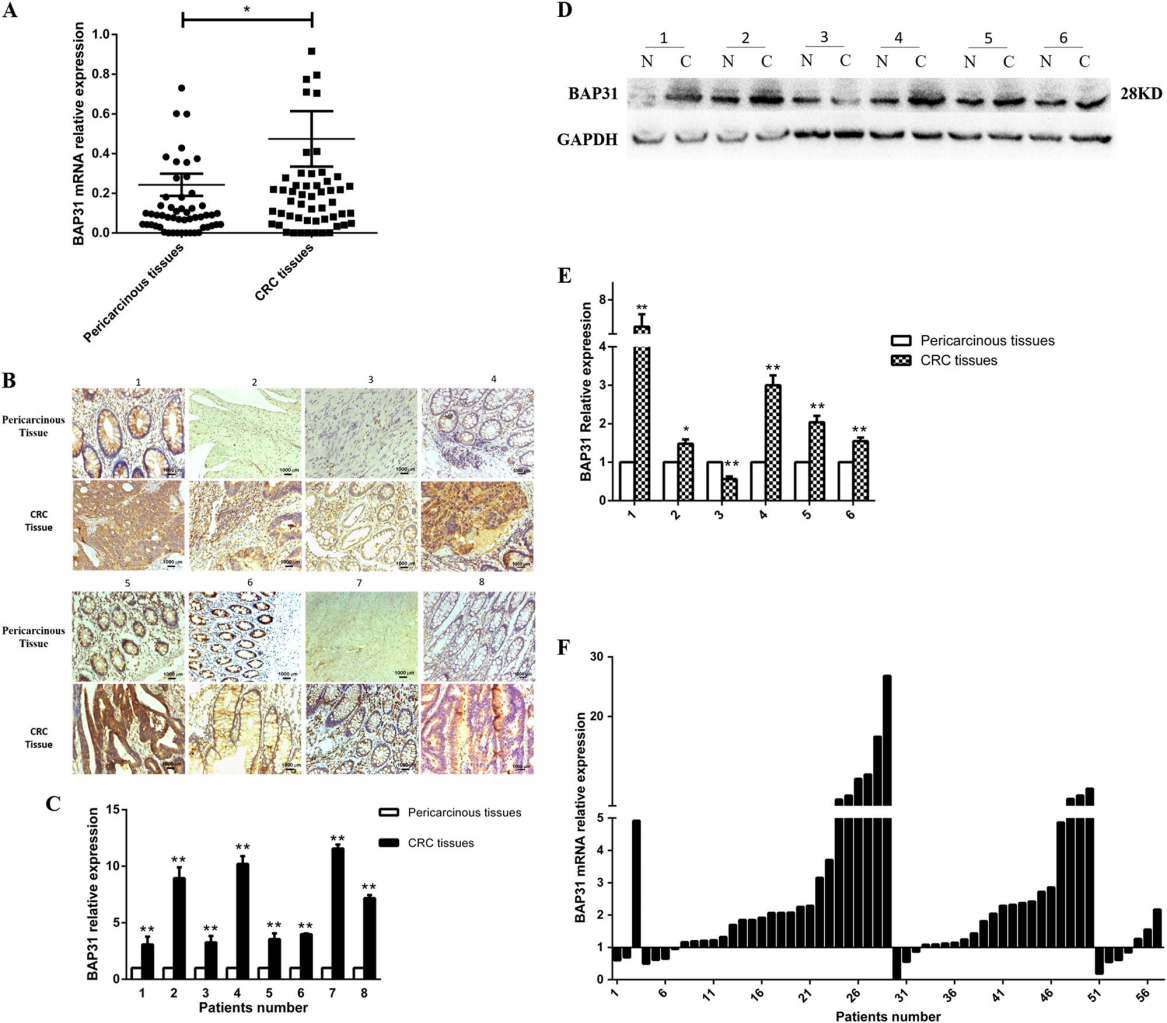
BAP31 expression was significantly elevated in CRC tissues compared to pericarcinous tissues. **a** The relative expression of BAP31 mRNA in CRC tissues of 57 cases compared to pericarcinous tissues as detected by real-time PCR (*P < 0.05). **b**, **c** The relative expression of BAP31 protein in CRC tissues compared to pericarcinous tissues as detected by immunohistochemistry (**P < 0.01; 1–6: six pairs of resected CRC specimens were randomly selected from stage II and stage III patients; samples 1–3 were in stage II, and samples 4–6 in stage III) and western blotting (**P < 0.01; N, pericarcinous tissues; C, colorectal cancer; 1–4: eight pairs of clinically resected CRC specimens randomly selected from stage II and stage III patients; samples 1–4 were in stage II, and samples 5–8 in stage III). **d**, **e** The relative expression of BAP31 protein in CRC tissues compared to pericarcinous tissues as detected by immunohistochemistry (scale bar, 50 μm; **P < 0.01). (**f**) The expression of BAP31 in CRC tissues of patients with different clinic stages: 1–3, stage I; 4–29, stage II; 30–50, stage III; 51–57, stage IV. CRC, colorectal cancer

To analyze the relative expression of BAP31 in CRC tissues with pericarcinous tissues, we performed real-time polymerase chain reaction (PCR) analysis in paired CRC and pericarcinous tissues in a cohort of 57 CRC patients. Results revealed that BAP31 mRNA expression in CRC tissues was significantly increased compared to matched pericarcinous tissues (Fig. 1a; *n* = 57, fold change: 4.87, *P < 0.05). There were 44 cases with up-regulated BAP31, accounting for 77.19% (Fig. 1a; 44/57). Immunohistochemical (IHC) staining results showed that BAP31 protein expression was significantly up-regulated in CRC tissues compared to matched pericarcinous tissues (Fig. 1b, c). Western blot analysis confirmed the IHC results (Fig. 1d, e; **P < 0.01). As shown in Table 1, the elevated BAP31 expression was significantly correlated with advanced clinical stages and distant metastasis of CRC (*P < 0.05), but not with other pathological parameters. Moreover, the upregulation of BAP31 was particularly obvious in clinical stage II and III CRC patients (Fig. 1f). There were 22 cases with up-regulated BAP31, accounting for 38.60% of the total cases, and 84.62% in 26 cases of stage II. There were 18 cases with up-regulated BAP31, accounting for 31.58% of the total cases and 85.71% in 21 cases of stage III (Table 1).

**Table 1:** Relationship between expression of BAP31 levels in colorectal cancer and clinic pathological features (*n* = 57, *P < 0.05)

Clinical characteristics	*N* (total %)	BAP31 relative expression		Statistic	*P* value
		Up-regulation *N* (total %) & (subject %)	Down-regulation *N* (total %) & (subject %)		
*Age (years)*					
≥ 56	34 (59.65)	28 (49.12) (82.35)	6 (10.53) (17.65)	1.274	0.339
< 56	23 (40.35)	16 (28.07) (69.57)	7 (12.28) (30.43)		
*Gender*					
Male	30 (52.63)	22 (38.60) (73.33)	8 (14.4) (26.67)	0.536	0.538
Female	27 (47.37)	22 (38.60) (81.48)	5 (8.77) (18.52)		
*Tumor location*					
Rectum	48 (84.21)	37 (59.65) (77.08)	11 (19.30) (22.92)	0.002	1.000
Colon	9 (15.79)	7 (12.28) (77.78)	2 (3.51) (22.22)		
*Differentiation*					
Low	10 (17.54)	6 (10.53) (60.00)	4 (7.02) (40.00)	2.036	0.213
Middle	47 (82.46)	38 (66.67) (80.85)	9 (15.79) (19.15)		
*Node status*					
N0	30 (52.63)	23 (40.35) (76.67)	7 (12.28) (23.33)	1.172	0.350
N1–N2	27 (47.37)	21 (36.84) (77.78)	6 (10.53) (22.22)		
*Distant metastasis*					
M0	50 (87.72)	41 (71.93) (82.00)	9 (15.79) (18.00)	5.344	0.041^*^
M1	7 (12.28)	3 (5.26) (42.86)	4 (7.02) (57.14)		
*Clinical stage*					
I	3 (5.26)	1 (1.75) (33.33)	2 (3.51) (66.67)	9.645	0.022^*^
II	26 (45.61)	22 (38.60) (84.62)	4 (7.02) (15.38)		
III	21 (36.84)	18 (31.58) (85.71)	3 (5.26) (14.29)		
IV	7 (12.28)	3 (5.26) (42.86)	4 (7.02) (57.14)		

### BAP31 was over-expressed in CRC cell lines

**Fig. 2:**
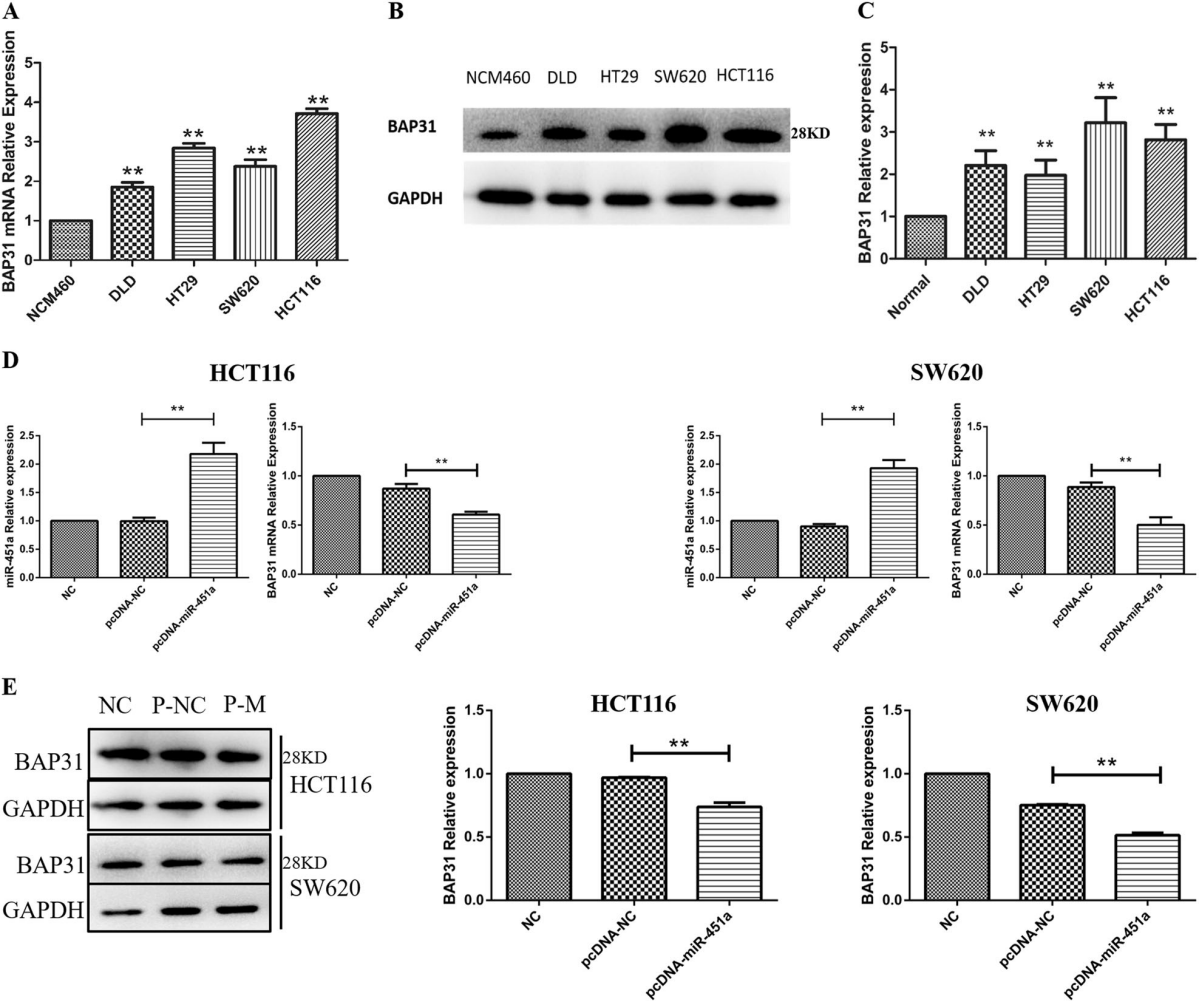
BAP31 mRNA and protein expression in different cell lines. **a** The relative expression of BAP31 mRNA in five CRC cell lines: DLD, HT29, SW620, HCT116, and normal colorectal epithelial cell line, NCM460 cells. **b**, **c** The relative expression of BAP31 protein in DLD, HT29, SW620, HCT116, and NCM460 cells (**P < 0.01). Over-expressing miR-451a reduced the expression of BAP31 mRNA and protein. **d** After transfection by pcDNA-miR-451a, the expression of miR-451a was increased and that of BAP31 mRNA was decreased in HCT116 cells and SW620 cells. **e** The expression of BAP31 protein in HCT116 and SW620 cells was decreased on over-expressing miR-451a (**P < 0.01; NC, untreated; P-NC, transferred pcDNA-NC; P-M, transferred pcDNA-miR-451a). CRC, colorectal cancer

We also detected the relative expression of BAP31 in five CRC cell lines compared to NCM460 cells. As shown in Fig. 2a, the BAP31 mRNA expressions in DLD, HT29, SW620, and HCT116 cells were 1.85, 2.84, 2.37, and 3.71 folds compared with those in NCM460 cells, respectively (**P < 0.01). As detected by western blotting, BAP31 protein expressions in DLD, HT29, SW620, and HCT116 cells were 1.97, 2.20, 3.22, and 2.81 folds compared with those in NCM460, respectively (Fig. 2b, c; **P < 0.01).

### MiR-451a inhibited the expression of BAP31 by binding to its 5’-UTR

To detect whether the up-regulated expression of miR-451a can directly decrease BAP31’s expression in CRC cell lines, plasmid pcDNA-miR-451a-over-expressing miR-451a was transfected into HCT116 and SW620 cells for 48 h. As detected by real-time reverse transcription-polymerase chain reaction (RT-PCR), miR-451a relative expression was increased by 83.95% in HCT116 and 87.91% in SW620 cells after the plasmid pcDNA-miR-451a was transfected into HCT116 and SW620 cells (Fig. 2d; **P < 0.01). BAP31 mRNA expression was decreased by 30.2% in HCT116 and 43.5% in SW620 cells, respectively (Fig. 2d; **P < 0.01). Over-expressing miR-451a, BAP31 protein expression was decreased by 23.8 and 31.6% in HCT116 and SW620 cells, respectively (Fig. 2e; **P < 0.01).

**Fig. 3:**
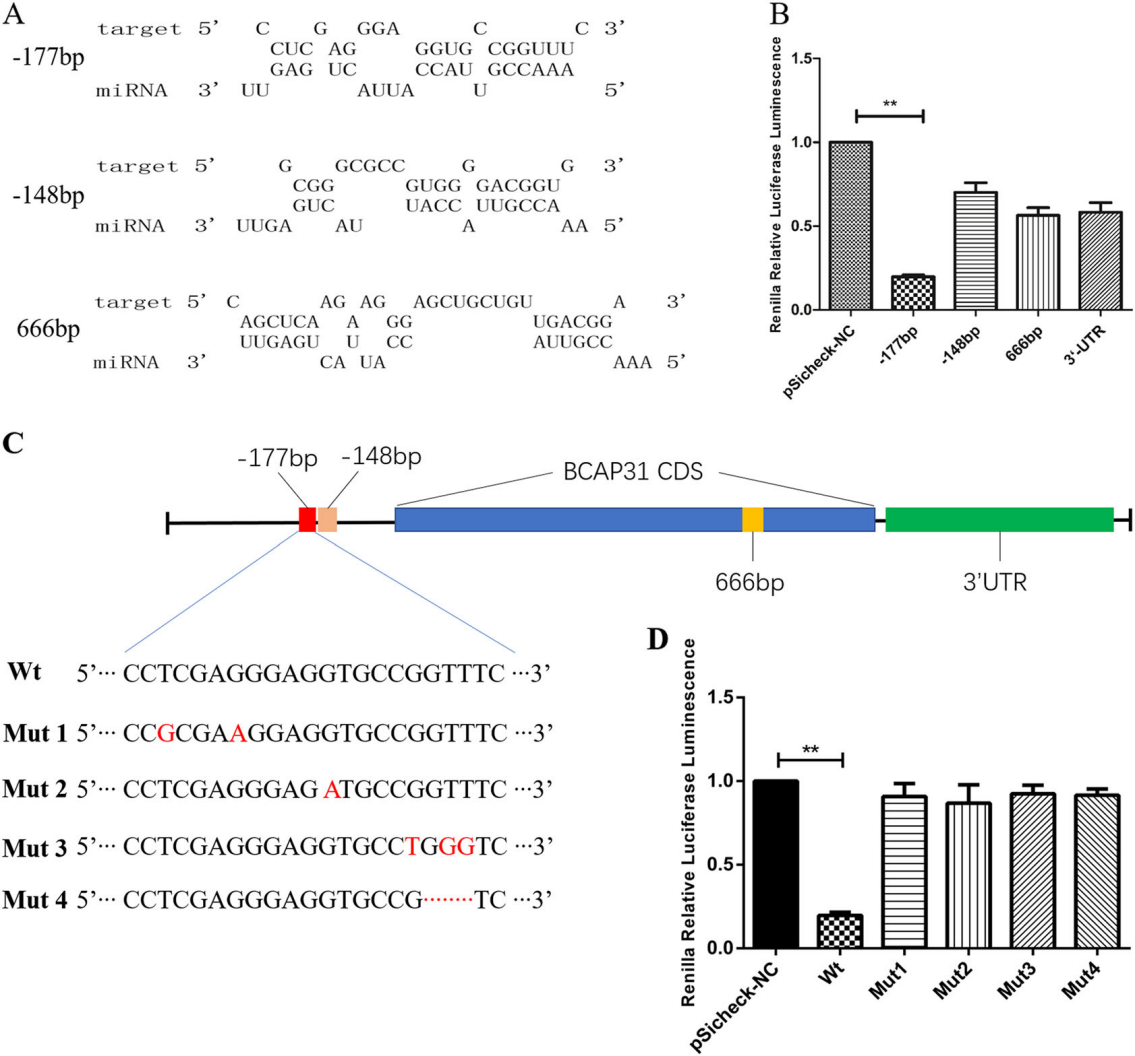
MiR-451a inhibited the expression of BAP31 by binding to its 5’-UTR. **a** The predicted miR-451a seed region in the upstream and the ORF of BAP31 gene. **b** When upstream 177 was blocked, the luciferase activity was significantly reduced by miR-451a (**P < 0.01). **c** The location of the predicted miR-451a seed region in BAP31 gene. The base changes and deletions in four mutations of BAP31 upstream 177 sequence. **d** The renilla luciferase relative activity in wild-type vector BAP31 upstream 177 site and mutations (Wt, wild-type; Mut1, mutation 1; Mut2, mutation 2; Mut3, mutation 3; Mut4, mutation 4). ORF, open reading frame

MiRNA usually binds with the 3’-UTR of its target genes, but rarely with the open reading frame (ORF) and 5’-UTR. MiRanda and TargetScan, the widely used microRNA target-prediction programs, were applied to predict BAP31 as miR-451a’s target. But we could not find any binding sites with miR-451a in BAP31 mRNA 3’-UTR. To elucidate the mechanisms of miR-451a down-regulating BAP31, RNAHybrid program was used to predict miR-451a’s binding sites of BAP31. Twenty binding sites were predicted by the program, among which three binding sites had the highest scores: BAP31 upstream 177, 148, and ORF 666 sites (Fig. 3a). They were cloned into pSicheck-2.0 vector. We also cloned the common mode of the 3’-UTR region of BAP31 into pSicheck-2.0 (Fig. 3c). These plasmids were co-transfected into 293T cells with pcDNA-miR451a. Firefly luciferase and renilla luciferase activity in each group was measured. Surprisingly, the renilla luciferase relative activity was decreased by 80.3, 30.1, 44.6, and 42.8% in vector BAP31 upstream 177 site, upstream 148 site, ORF 666 site sequence area, and BAP31 3’-UTR sequence area, respectively (Fig. 3b; **P < 0.01). Four mutations were performed in the miR-451a-binding sequence, including Mut1: T to G (-175 bp), G to A (-171 bp); Mut2: G to A (-166 bp); Mut3: G to T (-161 bp), T to G (-159 and -158 bp); and Mut4: deletions at -160, -159, and -158 bp (Fig. 3c). The four mutated sequences were cloned into pSicheck-2.0. These plasmids were also co-transfected into 293T cells with pcDNA-miR-451a. The renilla luciferase relative activity was decreased by 80.3, 15.89, 26.54, 7.54, and 8.43% in wild-type vector BAP31 upstream 177 site, mutation 1, mutation 2, mutation 3, and mutation 4, respectively (Fig. 3d; **P < 0.01). The results demonstrated that miR-451a can bind to the BAP31 mRNA 5’-UTR 177 site and cannot bind to the mutated sequences, which also supported BAP31 mRNA as a putative target of miR-451a.

### Over-expressing miR-451a or silencing BAP31 inhibited the proliferation of CRC cells

**Fig. 4:**
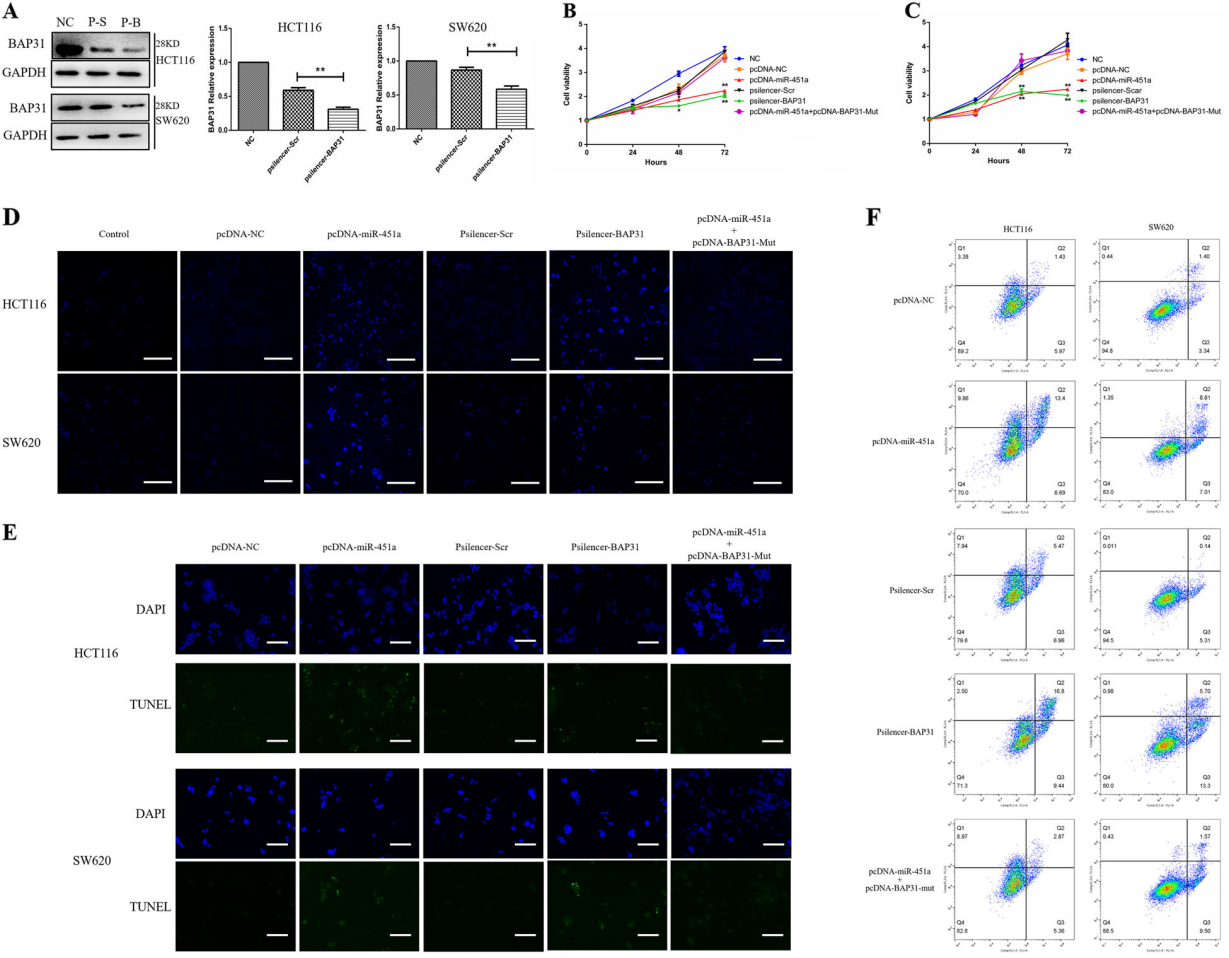
Over-expressing MiR-451a or silencing BAP31 suppressed the proliferation of CRC cells and increased the total apoptosis of CRC cells. **a** BAP31 protein relative expression was decreased in HCT116 and SW620 cells on silencing BAP31 (**P < 0.01; NC, untreated; P-S, transferred psilencer-Scr; P-B, transferred psilencer-BAP31). **b**, **c** Over-expressing miR-451a or silencing BAP31 exhibited significant inhibitory effects on the proliferation of HCT116 (**b**) and SW620 (**c**) cells compared to the control and untreated cells (NC). **d**, **e** Over-expressing miR-451a or silencing BAP31 induced significant apoptosis in HCT116 and SW620 cells compared to the control cells. The apoptotic cells were stained brightly blue by Hoechst staining (**d**; scale bar, 50 μm) and green fluorescence by TUNEL staining (**e**; scale bar, 25 μm). (**f**) As detected by flow cytometry, total apoptosis was increased by 12.69 and 13.81% when over-expressing miR-451a or silencing BAP31, respectively, in HCT116 cells. The total apoptosis increased by 10.88 and 13.55%, respectively, when over-expressing miR-451a or silencing BAP31 in SW620 cells. CRC, colorectal cancer

To further investigate the potential mechanism of down-regulation of BAP31 by over-expresseing miR-451a, we transfected pcDNA-miR-451a, pcDNA-NC, psilencer-BAP31, and control plasmid, pSilencer-Scr, into HCT116 and SW620 cells. To detect the efficiency of BAP31-silencing plasmid, western blotting was used to detect the expression of BAP31 when pSilencer-BAP31 and negative control plasmid, pSilencer-Scr, were transfected into HCT16 and SW620 cells for 48 h. As shown in Fig. 4a, BAP31 protein expression was decreased to 63.9 and 47.9% by over-expressing miR-451a in HCT116 and SW620 cells, respectively (**P < 0.01).

We further measured the proliferation of HCT116 and SW620 cells over-expressing miR-451a or silencing BAP31. To investigate whether over-expressing BAP31 with mutated miR-451a-binding sites can rescue the effect of over-expressing miR-451a, the full-length sequence of BAP31 was cloned and inserted into pcDNA-3.1 vector to obtain the construct pcDNA-BAP31. The miR-451a-binding sites were mutated (-161bp G to T, -159 bp and -158 bp T to G) to obtain the pcDNA-BAP31-Mut construct. HCT116 and SW620 cells were transfected with pcDNA-miR-451a, pcDNA-NC, psilencer-BAP31, pSilencer-Scr, and pcDNA-miR-451a + pcDNA-BAP31-Mut. As shown in Fig. 4b, c, over-expressing miR-451a or silencing BAP31 can inhibit the proliferation of HCT116 and SW620 cells at 48 h post transfection. After incubation for 72 h, the inhibitory effects were still obvious. The inhibition rates of over-expressing miR-451a and silencing BAP31 on HCT116 cells were 19.75 and 28.23% at 48 h, and 39.50 and 45.32% at 72 h, respectively. The inhibition rates of over-expressing miR-451a and silencing BAP31 in SW620 cells were 31.03 and 29.25% at 48 h, and 50 and 53.56% at 72 h, respectively. The inhibition of proliferation in HCT116 and SW620 cells induced by over-expressing miR-451a can be reversed by the over-expression of BAP31 with a mutated miR-451a-binding site.

### Over-expressing miR-451a or silencing BAP31 induced the apoptosis of CRC cells

To find the inhibition mechanism of over-expressing miR-451a and silencing BAP31 on the proliferation of CRC cells, apoptosis was measured by Hoechst staining, terminal dexynucleotidyl transferase(TdT)-mediated dUTP nick end labeling (TUNEL) staining, and flow cytometry. Hoechst staining and TUNEL staining results showed that nuclear fragmentation and apoptosis were significantly increased in HCT116 and SW620 cells when over-expressing miR-451a or silencing BAP31, which can be reversed by the overexpression of BAP31 with a mutated miR-451a-binding site when over-expressing miR-451a (Fig. 4d, e). Flow cytometry results showed that the total apoptosis was increased by 12.69% in HCT116 cells over-expressing miR-451a, in which the early and late apoptosis increased by 0.72 and 11.97%, respectively. The total apoptosis in HCT116 cells silencing BAP31 was increased by 13.81%, in which the early and late apoptosis increased by 2.48 and 11.33%, respectively. The early apoptosis in HCT116 cells over-expressing BAP31 with a mutated miR-451a-binding site when over-expressing miR-451a did not have significant changes, and the late apoptosis increased by 1.44% (Fig. 4f). The total apoptosis of SW620 cells over-expressing miR-451a was increased by 10.88%, including a 3.67% increased early apoptosis and 7.21% increased late apoptosis. Apoptosis in SW620 cells silencing BAP31 increased by 13.55%, in which early apoptosis increased by 7.99% and late apoptosis increased by 5.56%, respectively. The total apoptosis in SW620 cells over-expressing BAP31 with a mutated miR-451a-binding site when over-expressing miR-451a was increased by 6.63%, in which the early and late apoptosis increased by 6.16 and 0.17%, respectively (Fig. 4f).

### Over-expressing miR-451a or silencing BAP31 induced ERS by up-regulating ERS-related proteins and increasing the cytoplasmic calcium concentration in CRC cells

**Fig. 5:**
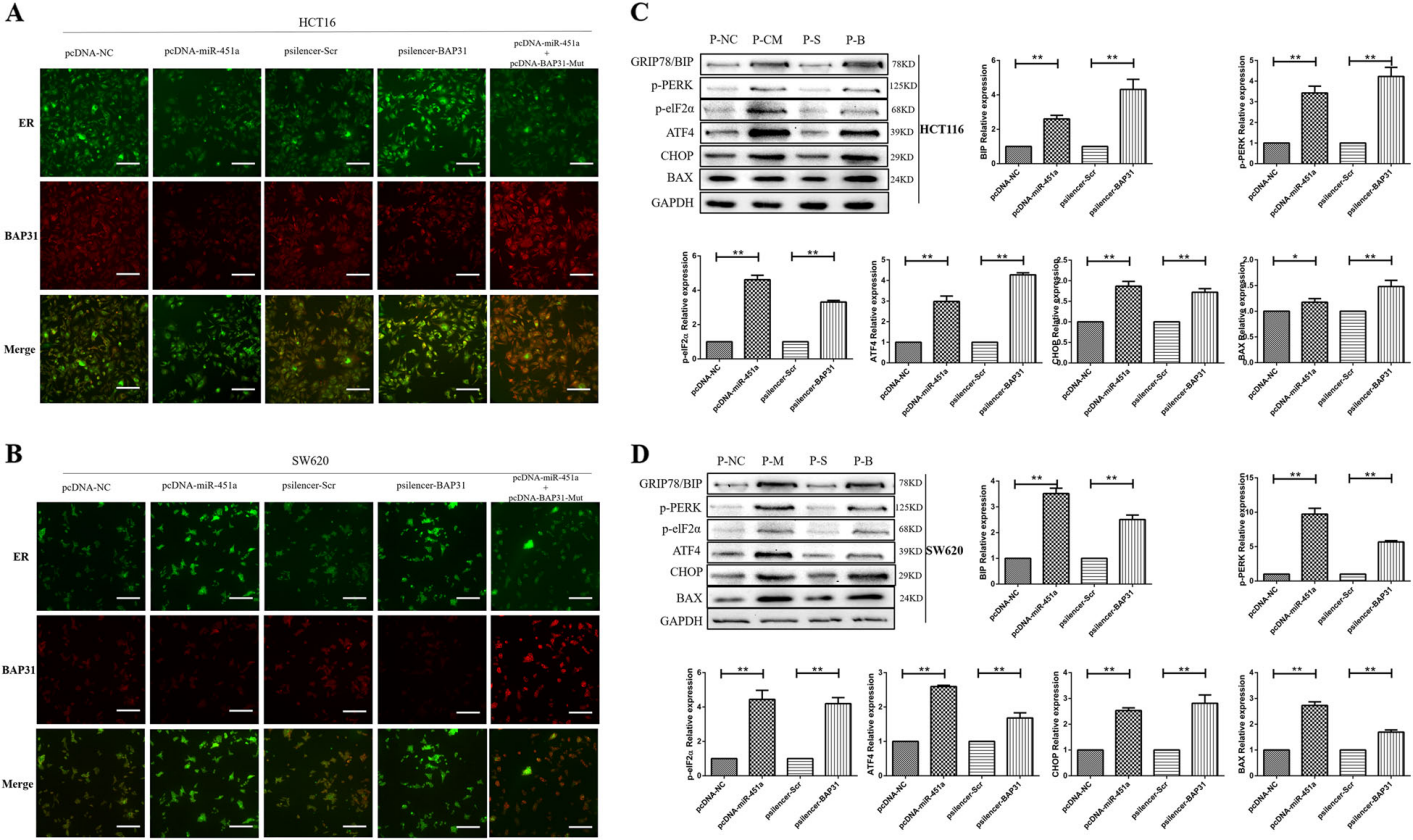
Expression and cellular location of BAP31, and the expression of ERS-associated proteins when over-expressing miR-451a or silencing BAP31. **a**, **b** ER-specific fluorescent dye ER-tracker was used to detect ER morphology. BAP31 fluorescent-conjugated antibody was used to detect BAP31 expression and cellular location. In HCT116 (**a**) and SW620 (**b**) cells, the normal morphology and structure of endoplasmic reticulum cannot be observed, while a lot of concentrated or vacuole-like structures were obvious (scale bar, 25 μm). **c**, **d** The expressions of ERS-associated proteins GRP78/BIP, BAX, p-PERK, p-elF2α, ATF4, and CHOP in HCT116 (**c**) and SW620 cells (**d**) were significantly increased on over-expressing miR-451a or silencing BAP31 (**P < 0.01, *P < 0.05; P-NC, transferred pcDNA-NC; P-M, transferred pcDNA-miR-451a; P-S, transferred psilencer-Scramble; P-B, transferred psilencer-BAP31). ERS, endoplasmic reticulum stress

**Fig. 6:**
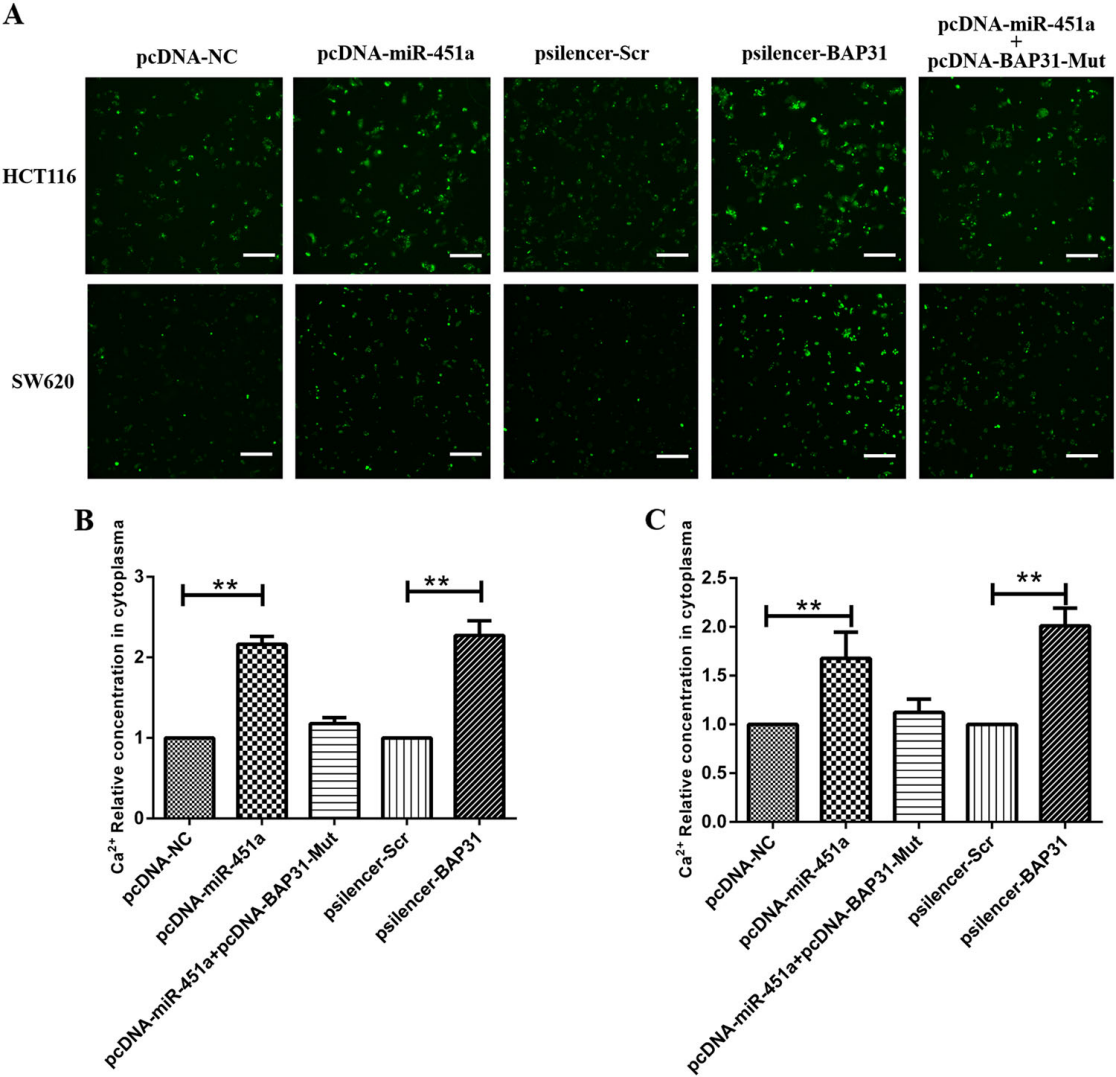
Over-expressing miR-451a or silencing BAP31 increased intracellular calcium concentration. **a** Cytosolic calcium-specific fluorescent dye Fluo-4AM was added into the cell culture medium to a final concentration of 10 μM to detect calcium ions in the cytoplasm 48 h after transfection with pcDNA-miR-451a, pcDNA-NC, psilencer-BAP31, and psilencer-Scr in HCT116 and SW620 cells (scale bar, 50 μm). **b** Cytosolic calcium concentration was 1.67- and 2.01-fold higher in HCT116 cells over-expressing miR-451a or silencing BAP31, respectively. **c** Cytoplasmic calcium concentration was 2.08- and 2.27-fold higher in SW620 cells over-expressing miR-451a or silencing BAP31, respectively ( × 200)

Since BAP31 was located in endoplasmic reticulum, we further detected the morphology and protein-expression changes in endoplasmic reticulum after over-expressing miR-451a or silencing BAP31. Immunofluorescence results showed that BAP31 protein expressions in HCT116 and SW620 cells over-expressing miR-451a or silencing BAP31 were both reduced and remained localized in the endoplasmic reticulum. And most of the endoplasmic reticulum lost the normal morphology and structure, and appeared concentrated or acquired a vacuolar structure. When over-expressing BAP31 with a mutated miR-451a-binding site with miR-451a over-expression, the expression of BAP31 on the endoplasmic reticulum and the morphology and structure of endoplasmic reticulum had no significant changes compared to the control (Fig. 5a, b). When ERS occurs, the expression of ERS-related proteins also increases to accelerate the processing of unfolded proteins and thereby reduce ERS; so we detected the relative expression of the ERS-related protein, GRP78/BIP, and the downstream PERK/elF2α/ATF4/CHOP signaling pathway. The results showed that when over-expressing miR-451a or silencing BAP31 in HCT116 and SW620 cells, the expressions of GRP78/BIP were all increased to different extents (Fig. 5c, d). In the PERK/elf2α/ATF4/CHOP signaling pathway, the relative expressions of phosphorylated PERK, phosphorylated elF2α, ATF4, and CHOP were all increased to different extents. When ERS occurs, the first response is to release the endoplasmic reticulum calcium ion into the cytosol. So we detected BAX, an endoplasmic reticulum calcium releasing-associated protein, and the cytoplasmic calcium concentration in HCT116 and SW620 cells when over-expressing miR-451a or silencing BAP31. The results showed that the relative expression of BAX was significantly increased compared to the control cells (Fig. 5c, d). And the cytoplasmic calcium concentration in HCT116 and SW620 cells over-expressing miR-451a or silencing BAP31 was also increased (Fig. 6a). Cytosolic calcium concentration was 1.67-fold higher in HCT116 cells over-expressing miR-451a than in the control group. When silencing BAP31 in HCT116 cells, the cytoplasmic calcium concentration was 2.01-fold higher than that in the control group (Fig. 6b). Cytosolic calcium concentration was 2.08-fold higher in SW620 cells over-expressing miR-451a than that in the control group, while silencing BAP31 in SW620 cells increased the cytoplasmic calcium concentration by 2.27 fold (Fig. 6c). But the cytoplasmic calcium concentration did not significantly change when over-expressing BAP31 with a mutated miR-451a-binding site with miR-451a over-expressed both in HCT116 and SW620 (Fig. 6b, c).

### Over-expressing miR-451a or silencing BAP31 inhibited tumor growth in vivo

**Fig. 7:**
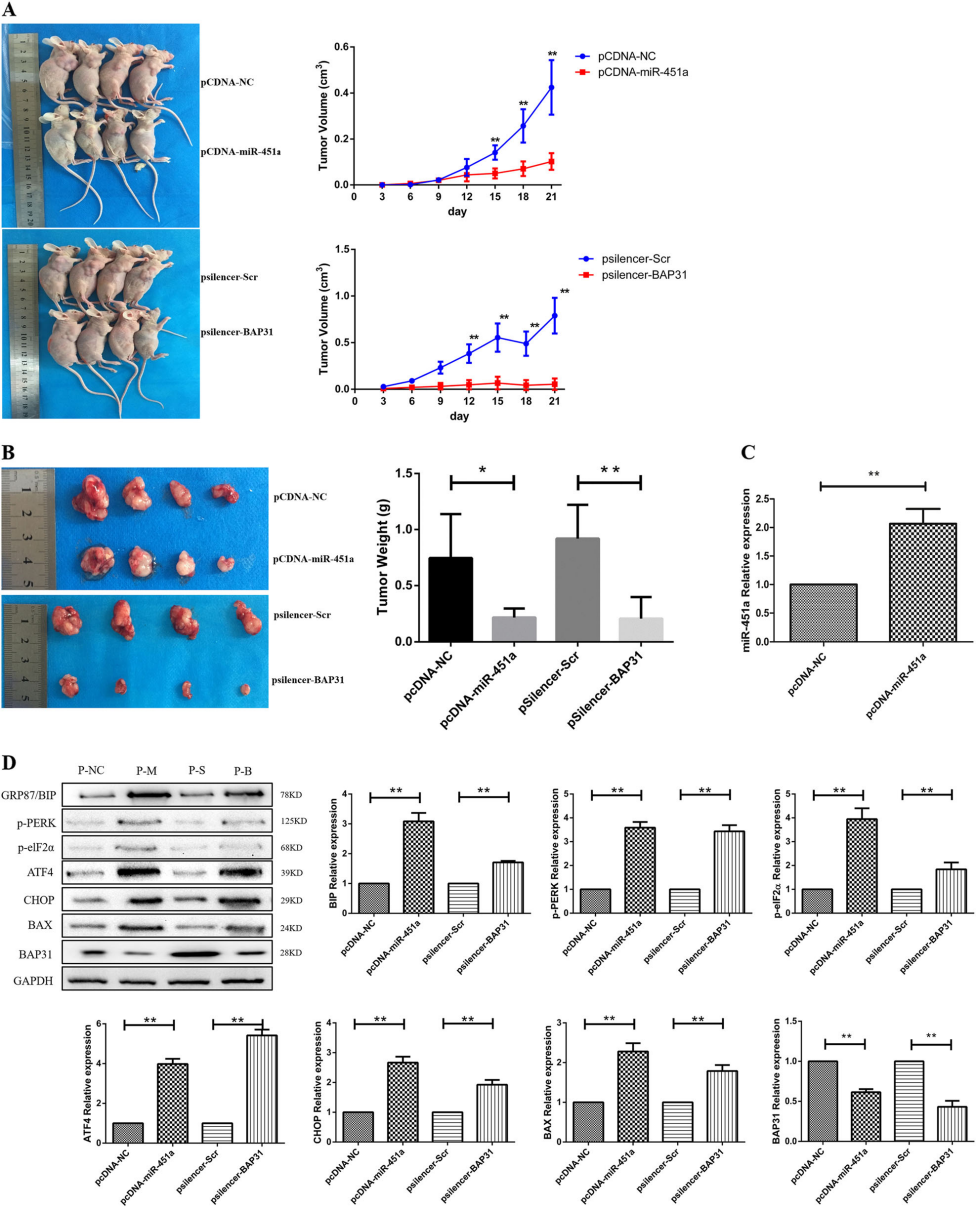
Over-expressing miR-451a or silencing BAP31 inhibited tumor growth in vivo and the relative expression of miR-451a, BAP31, and ERS-associated proteins in CRC tissues of different groups of mice. Twenty 5-week-old BALB/c-nu male mice were assigned into four groups: pcDNA-NC control group, pcDNA-miR-451a group, psilencer-Scr control group, and psilencer-BAP31 group, each group with five mice. As many as 8 × 10^6^ cancer cells were subcutaneously injected into these mice. The weight of the mice and tumor volumes were determined every 3 days. The mice were sacrificed when administered for 21 days. The tumors were collected and weighed. **a** Tumor xenograft volume was smaller in nude mice treated with plasmids over-expressing miR-451a or silencing BAP31 than that in the control group. **b** The body weights of tumor xenografts were also lower than those of the control group (*P < 0.05, **P < 0.01). **c** The relative expression of miR-451a in xenografts administered with pcDNA-miR-451a was higher than that of the control group (**P < 0.01). **d** The relative expression of BAP31 was lower in pcDNA-miR-451a and psilencer-BAP31 groups than those in control groups (**P < 0.01). The expressions of ERS-associated proteins were higher in pcDNA-miR-451a and psilencer-BAP31 groups than those in control groups (**P < 0.01,*P < 0.05). CRC, colorectal cancer; ERS, endoplasmic reticulum stress

To further confirm the above findings, we established CRC xenografts in nude mice to determine the effects of miR-451a and BAP31. Tumor growth curve showed that the tumor growth rate was inhibited when over-expressing miR-451a or silencing BAP31 in vivo (Fig. 7a). Tumor weights of pcDNA-miR-451a and pSilencer-BAP31 groups were significantly decreased to 18.17 and 12.51% of the control group, respectively (Fig. 7b; **P < 0.01). Real-time PCR results showed that the expression of miR-451a in the tumor tissues of pcDNA-miR-451a group was significantly higher than that in the control group (Fig. 7c; **P < 0.01). Western blot results also showed that BAP31 expression was inhibited in pcDNA-miR-451a and pSilencer-BAP31 groups (Fig. 7d; **P < 0.01). The relative expressions of GRP78/BIP, p-PERK, p-elF2α, ATF4, CHOP, and BAX were increased in pcDNA-miR-451a and pSilencer-BAP31 groups compared with the control group, respectively (Fig. 7d; **P < 0.01).

## Discussion

Our results showed that the expression of BAP31 in CRC tissues was significantly increased, while the expression of miR-451a in CRC tissues was obviously down-regulated. We found that BAP31 relative expression levels were correlated with advanced clinical stage and distant metastasis of CRC. We also found that the expression of BAP31 in clinical stage II and III cases was most obviously increased, in which the expression of miR-451a was obviously decreased. We also found that the expressions of BAP31 mRNA and protein were up-regulated in the CRC cell lines, HCT116, HT29, SW620, and DLD, compared with normal colonic epithelial cells, NCM460. In our previous experiments, HCT116 and SW620 cells had a relatively lower expression of miR-451a among HCT116, SW620, HT29, SW480, and DLD cell lines. We also demonstrated that after the down-regulation of miR-451a, the expression of BAP31 was increased in our suppression subtractive hybridization library^15^. And now, the BAP31 relative expression was also higher in these cell lines, which suggested a potential negative correlation between the expression of miR-451a and BAP31. Our in vitro and in vivo results demonstrated that BAP31 plays a very important role in the carcinogenesis of CRC under the direct regulation of miR-451a.

The classic pattern of miRNA-regulating target genes is to bind to the 3’-UTR of its target genes. So most of the miRNA target gene-prediction softwares are based on the 7–6 bases in the 3’-UTR region of the target genes, which are strictly paired to miRNA seed sequence^23^. This prediction model does provide convenience for the research of a large number of target genes of miRNA. However, more and more studies have found that miRNA may regulate target genes by binding to the 5’-UTR or ORF region^24–26^. The 3’-UTR classical prediction model showed that the binding between miR-451a and BAP31 3’-UTR regions was not strong. Our dual-luciferase reporter experiments demonstrated that miR-451a could directly regulate BAP31 by binding to its 5’-UTR instead of 3’-UTR. When miR-451a-binding sites were mutated, miR-451a cannot bind to the mutated sequences any longer. Our study does provide new evidence for understanding the mechanisms of miRNA-regulating target genes.

BAP31 has three transmembrane domains on the endoplasmic reticulum. Its N terminus is located in the endoplasmic reticulum lumen, and C terminus in the cytoplasm, mediating protein–protein interactions^20,27–29^. The relative expression of BAP31 was dramatically up-regulated in malignant human melanoma and primary hepatocellular carcinoma when compared with normal human tissues. However, the significance of its over-expression and its function remains unclear^27,30,31^. We also found that over-expressed miR-451a or silenced BAP31 could significantly inhibit the proliferation of HCT116 and SW620 cells, which resulted from increasing ERS-associated protein expressions and calcium ions releasing from the endoplasmic reticulum into the cytoplasm. These mechanisms induced ERS and increased the apoptosis of CRC cells.

The endoplasmic reticulum is widely distributed within the cell and plays a very important role. In the endoplasmic reticulum, when the expression of the built-in chaperone proteins changes, the cytosolic calcium concentration decreases. Then, chemically toxic substances will accumulate and stimulate oxygen stressing^18,32–35^. When a large number of unfolded or misfolded proteins accumulate in the endoplasmic reticulum, the normal physiological function will be changed. This phenomenon is known as ERS^36,37^. ERS primarily acts through activating IRE-1, which is a significant event in andrographolide-induced CRC cell death^38^.

**Fig. 8:**
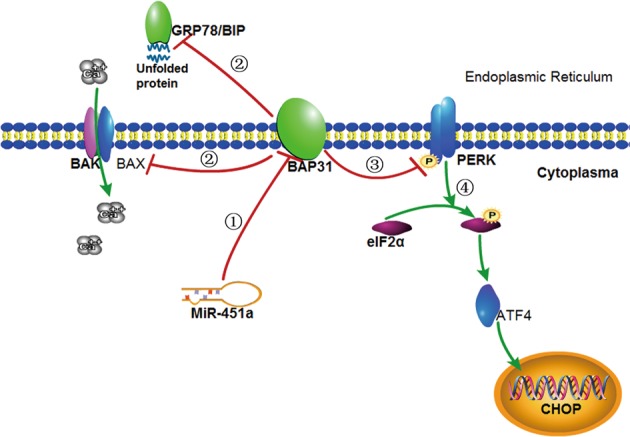
**MiR-451a can suppress BAP31 expression and induce apoptosis through ERS, which promote the carcinogenesis of CRC**

From the above, we have the following speculation for carcinogenesis of CRC (Fig. 8). Decreased miR-451a expression in CRC induces the over-expression of BAP31, which increases expressions of PERK, GRP78/Bip, and BAX. We have validated it in the present study. GRP78/BiP, a major chaperone protein involved in protein folding and transporting, promotes proper protein conformation. PERK binds to GRP78/BiP when the endoplasmic reticulum is under normal circumstances^39,40^. Under conditions of hypoglycemia, hypoxia, and low calcium, ERS will be induced, which will promote the releasing of GRP78 from these ER membrane proteins and binding to misfolded proteins^41^. GRP78/Bip regulates cell proliferation, apoptosis, invasion, metastasis, as well as angiogenesis through activating a series of signal transduction pathways^42^. PERK is an endoplasmic reticulum type I transmembrane protein belonging to Ser/Thr protein kinase. It is activated and self-phosphorylated on the endoplasmic reticulum when GRP78/Bip disassociates from the GRP78/Bip/PERK complex. Activated PERK will result in the phosphorylation of downstream elF2α to prevent more protein production. Meanwhile, the phosphorylation of elF2α activates the elF2α/ATF4/CHOP signaling pathway^43–45^. In addition, BAX and BAK proteins can regulate the calcium leak from the endoplasmic reticulum^46^. BAX on the endoplasmic reticulum and mitochondria can be an important starting apoptotic signal protein. When ERS occurs, the first response is to release the endoplasmic reticulum calcium ion into the cytosol. The cytosolic calcium concentration will be increased and will then activate mitochondrial apoptosis pathway^35,46^.

In conclusion, the present study demonstrated that BAP31 is the target of miR-451a. Over-expressing miR-451a or BAP31 can inhibit proliferation and induce apoptosis in CRC through inducing ERS.

## Materials and methods

### Patients and tissue samples

The CRC samples and pericarcinous tissues were surgically resected and quickly stored in liquid nitrogen from 57 CRC patients from the Department of Surgery, West China Hospital, Sichuan University, from November 2014 to January 2016. The pericarcinous tissues, which were away from tumors at least by 5 cm, did not contain cancer cells, which usually appeared inflammatory and fibrotic. Pathological diagnosis was made by two pathologists independently according to the World Health Organization classification^47^. Tumors were staged according to the TNM Classification of Malignant Tumors (TNM)^48^. None of the patients underwent chemotherapy before surgical resection. This study was approved by the Ethics Committee of West China Hospital (No. K2016041). Written informed consent was obtained from patients before sampling.

### Real-time RT-PCR

Total RNA was extracted from tissues with Trizol according to the manufacturer’s instructions (Takara, Dalian, China). Its concentration and purity were determined using BioPhotometer (Eppendorf, Hamburg, Germany). Its integrity was checked with 1% denatured agarose gel. First-strand cDNA was synthesized from 2 µg of total RNA using 100U of Moloney murine leukemia virus reverse transcriptase (Promega, Shanghai, China) in a 10 μl reaction mixture containing 10 U of RNase inhibitor and 1 µl of 10 mM reverse transcription primers as follows.

miR-451a forward primer:

5’-ACACTCCAGCTGGGAAACCGTTACCATTAC-3’;

miR-451a reverse primer:

5’-CTCAACTGGTGTCGTGGAGTCGGCAATTCAGTTGAGCTTACAG-3’.

U6 forward primer: 5’-CTCGCTTCGGCAGCAC-3’;

U6 reverse primer: 5’-AACGCTTCACGAATTTGCG-3’.

BAP31 forward primer: 5’-CTGCTGTCCTTCCTGCTTAG-3’;

BAP31 reverse primer: 5’-CTCCTGTTCTCTTCCTCCAAC-3’.

GAPDH forward primer: 5’-AGAAGGCTGGGGCTCATTTGC-3’;

GAPDH reverse primer: 5’-ACAGTCTTCTGGGTGGCAGTG-3’.

The real-time RT-PCR was run with AceQ^TM^ qPCR SYBR Green Master Mix (Vazyme, Nanjing, China) in a final volume of 20 μl, containing 10 μl of the master mix, each with 0.4 μl of 10 µM forward and reverse primers, and 2 μl of cDNA. The thermal cycling program was as follows: an initial heating at 95 °C for 5 min, followed by 35 cycles of 10 s at 95 °C, and 60 °C for 30 s. Expression of miR-451a was normalized to the internal U6 levels. The mRNA of BAP31 expression was normalized to the mRNA of GAPDH. Each sample was run in triplicate, and the threshold cycle numbers (Ct) were averaged. Melting curve analysis was performed by increasing the temperature from 65 to 95 °C in 0.1 °C/s increments for each fluorescence reading using the CFX96 Touch™ qPCR system (Bio-Rad, California, USA).

### Cell culture

Five CRC cell lines, HCT116, HT29, SW480, SW620, DLD, and a normal colonic epithelial cell line NCM460, and human 293T embryonic kidney cell lines were obtained from American Type Culture Collection (ATCC, Manassas, VA, USA). They were cultured in Dulbecco’s modified Eagle's medium containing 10% fetal bovine serum, 100 U/ml penicillin, and 100 µg/ml streptomycin, and maintained in a humidified incubator with 5% CO_2_ at 37 °C.

### Construction of vector over-expressing miR-451a

The constructed vector over-expressing miR-451a was transfected into HCT116 and SW620 cells for other experiments. The full length of pre-miR-451a amplified by pcDNA-miR-451aF and pcDNA-miR-451aR was cloned and inserted into the vector pcDNA-3.1 ( + ) and the empty vector pcDNA-NC as the negative control. The primers used are as follows:

pCDNA-miR-451aF:

5’-CGCGGATCCAGCCTGACAAGGACAGG-3’;

pCDNA-miR-451aR:

5’-CCGCTCGAGCCCACCCCTGCCTTGTTTG-3’.

### Dual-luciferase reporter vector construction

The sites of miR-451a binding with BAP31 gene were predicted by the online software tool RNA hybrid, in http://bibiserv.techfak.uni-bielefeld.de/rnahybrid/ website. To construct dual-luciferase reporter vector, the primers designed were as follows:

### BAP31-177

Forward primer: 5’-CCGCTCGAGGTTTCTCTCCGCGGGCG-3’;

reverse primer: 5’-ATAAGCGGCCGCGAGGCGGGCGCTCTG-3’.

### BAP31-148

Forward primer: 5’-CCGCTCGAGTGCGCTTGGTCCCGAATC-3’;

reverse primer: 5’-ATAAGCGGCCGCGGAGAGAAACCGGCACCTC-3’.

### BAP31-666

Forward primer: 5’-CCGCTCGAGGGAGAGTGCTAGTGAGGCG-3’;

reverse primer: 5’-ATAAGCGGCCGCTTCTCTTCCTCCAACTTCACCTC-3’.

### BAP31-3'-UTR

Forward primer: 5’-CCGCTCGAG GAAGAGTAAGGGCCTCCTTC-3’;

reverse primer: 5’-ATAAGCGGCCGCTTGCCCTCAAGAGAAACTCC-3’.

### Mutation 1

Forward primer: 5’-AGGCCGCTGGATCCGCGAAGGAGGTGCCGGTTTC-3’;

reverse primer: 5’-GAAACCGGCACCTCCTTCGCGGATCCAGCGGCCT-3’.

### Mutation 2

Forward primer: 5’-CTGGATCCTCGAGGGAGATGCCGGTTTCTCTCC-3’;

reverse primer: 5’-GGAGAGAAACCGGCATCTCCCTCGAGGATCCAG-3’.

### Mutation 3

Forward primer: 5’-TCGAGGGAGGTGCCTGGGTCTCTCCGCGGC-3’;

reverse primer: 5’-GCCGCGGAGAGACCCAGGCACCTCCCTCGA-3’.

### Mutation 4

Forward primer: 5’-CGAGGGAGGTGCCTTTCTCTCCGCGGCC-3’;

reverse primer: 5’-GGCCGCGGAGAGAAAGGCACCTCCCTCG-3’.

BAP31-177, -148, -666, and -3’-UTR sequences were cloned by PCR using the cDNA extracted from HCT116 cells as the template with primers and inserted into the pSicheck-2 vector to obtain the constructed vectors pSicheck-BAP31-148, pSicheck-BAP31-177, pSicheck-BAP31-666, and pSicheck-BAP31-3'-UTR. pSicheck-NC is the pSicheck-2 vector without any insertion. The miR-451a-binding site in -177 region was mutated using a site-directed gene mutagenesis kit (Beyotime, Beijing, China) according to the manufacturer’s instructions to obtain the pSicheck-BAP31-177-Mut (1–4) construct from the construct pSicheck-BAP31-177. The constructed vector pcDNA-miR-451a was transfected into 293T cells for dual-luciferase assay. All the luciferase reporter vectors were constructed according to the following combinations: Blank, pCDNA-NC + pSicheck-NC, pCDNA-miR451a + pSicheck-NC, pCDNA-miR451a + pSicheck-BAP31-148, pCDNA-miR451a + pSicheck-BAP31-177, pCDNA-miR451a + pSicheck-BAP31-666, pCDNA-miR451a + pSicheck-BAP31-3UTR, pCDNA-NC + pSicheck-BAP31-148, pCDNA-NC + pSicheck-BAP31-177, pCDNA + pSicheck- BAP31-666, pCDNA-NC + pSicheck-BAP31-3UTR, pCDNA-miR451a + pSicheck-BAP31-177-Mut1, pCDNA-miR451a + pSicheck-BAP31-177-Mut2, pCDNA-miR451a + pSicheck-BAP31-177-Mut3, pCDNA-miR451a + pSicheck-BAP31-177-Mut4, pCDNA-NC + pSicheck-BAP31-177-Mut1, pCDNA-NC + pSicheck-BAP31-177-Mut2, pCDNA-NC + pSicheck-BAP31-177-Mut3, pCDNA-NC + pSicheck-BAP31-177-Mut4. They were transfected into 293T cells when cells reached 80% confluence. The amount of constructed or untreated pSicheck vector was twice over that of the constructed vector pcDNA-miR-451a-co-transfected 293T cells. After 48 h co-incubation, the cells were collected, washed with phosphate-buffered saline (PBS), and added into 400 μl passive lysis buffer lysate. The vitalities of a small amount of 20 μl cell lysate and 100 μl luciferase test reagent II were mixed. Firefly luciferase with fluorescence illumination was immediately detected. After testing the firefly luciferase activity, 100 μl of Stop & Glo^TM^ reagent was added into the fluorescent illumination intensity meter tube. Firefly luciferase activation was quenched and Renilla luciferase reaction was activated immediately. To deduct the internal-reference firefly fluorescence emission intensity, the relative Renilla fluorescence intensity of the rest of the group was analyzed compared to pCDNA + pSicheck-NC, the negative control group.

### Construction of BAP31-over-expressing mutation vector

The full length of BAP31 amplified by primers pcDNA-BAP31F and pcDNA-BAP31R was cloned and inserted into the vector pcDNA-3.1 ( + ) to obtain the pcDNA-BAP31 construct. The miR-451a-binding site in -177 region was mutated using a site-directed gene mutagenesis kit (Beyotime, Beijing, China) according to the manufacturer’s instructions to obtain the pcDNA-BAP31-Mut construct from the construct pcDNA-BAP31 by primers pcDNA-BAP31-MutF and pcDNA-BAP31-MutR. The pcDNA-miR-451a and pcDNA-BAP31-Mut constructs were co-transfected into HCT116 and SW620 to detect if the phenotype of miRNA over-expression is reversed by the over-expression of BAP31 with a mutated miRNA-binding site. The primers used are as follows:

pCDNA-BAP31F:

5’-CGCGGATCCCTTCTGAGGATACTGCGTCTA-3’;

pCDNA-BAP31R:

5’-CGGAATTCCTTACTCTTCCTTCTTGTCCAT-3’.

pCDNA-BAP31-Mut F:

5’-TCGAGGGAGGTGCCTGGGTCTCTCCGCGGG-3’;

pCDNA-BAP31-Mut R:

5’-CCCGCGGAGAGACCCAGGCACCTCCCTCGA-3’.

### Small-interference RNA construction

pSilencer 4.1-CMV neo vector was used for the expression of siRNA in HCT116 and SW620 cells. The gene-specific insert specified a 19-nucleotide sequence corresponding to the nucleotides (619 site) downstream of the transcription start site of mBAP31 (sense and antisense), which was separated by a 9-nucleotide non-complementary spacer from the reverse complement of the same 19-nucleotide sequence. This vector was referred to as a silencing vector, psilencer-BAP31. A control vector, pSilencer-Scr, was constructed using a 19-nucleotide sequence (Scramble sense and Scramble antisense) with no significant homolog to any mammalian gene sequence and therefore served as a negative control.

### BAP31 sense

5’**-**GATCCGTGGGAAACCTTCTAGCAATTCAAGAGATTGCTAGAAGGTTTCCCACAGA-3’;

### BAP31 antisense

5’**-**AGCTTCTGTGGGAAACCTTCTAGCAATCTCTTGAATTGCTAGAAGGTTTCCCACG-3’.

### Scramble sense

5’**-**GATCCGATTTAGGACCGCATATCTTTCAAGAGAAGATATGCGGTCCTAAATCATA-3’;

### Scramble antisense

5’**-**AGCTTTAGATTTAGGACCGCATATCTTCTCTTGAAAGATATGCGGTCCTAAATCG-3’.

### Transfection

HCT116 and SW620 cells were seeded in 96-well plates or six-well plates and incubated in 5% CO_2_ at 37 °C. When the cells reached 80% confluence, pcDNA-NC, pcDNA-miR451a, pSilencer-Scr, pSilencer-BAP31, and pcDNA-miR-451a + pcDNA-BAP31-Mut were transfected into the cells with Lipofectamine™ 2000 (Invitrogen, Carlsbad, USA) according to the manufacturer’s instructions.

### MTT assays

At 0, 24, 48, 72, 96, and 120 h after transfection, 20 μl of 3-(4,5-dimethyl-2-thiazolyl)-2,5-diphenyl-2-H-tetrazolium bromide (MTT) solution (5 mg/ml) was added into each well, three wells for each time point. Then, the culture medium was discarded after 4 h. One hundred and fifty microliters of dimethyl sulfoxide was added to each well and gently shaken in darkness for 10 min until the crystal fully dissolved. Absorbance (OD) at 570 nm was detected in the detection microplate reader.

### Hoechst staining

Cells were fixed with 4% formaldehyde for 20 min and gently washed to remove the fixative solution. At the cellular climbing film, a small amount of the hoechst33342 dye was dropped to cover the sample. Then, the cells were incubated at room temperature for 3–5 min. The hoechst33342 dye solution was removed. Cells were washed twice with PBS and observed under fluorescence microscopy after having mounted.

### TUNEL staining

Cells were washed with PBS and fixed with 4% formaldehyde for 20 min. In order to gently remove the fixative solution, the cells were washed with PBS. Triton X-100 (0.3%) was added to the cells for incubation for 5 min at room temperature and then washed with PBS. The apoptotic cells were analyzed by an in situ one-step TUNEL apoptosis assay kit (Beyotime, Jiangsu, China), following the procedure specified by the manufacturer. DNA fragmentation of the nuclei in the injured areas was stained. Apoptotic cells showed green fluorescence.

### Immunofluorescence

HCT116 and SW620 cells were fixed with 4% formaldehyde for 48 h, washed with PBS containing 2% triton, blocked for 1 h at room temperature with bovine serum, and incubated at 4 °C with BAP31 antibody (Proteintech, Rosemont, USA) overnight. Then cells were washed with PBS and incubated at room temperature for 1 h with the conjugated secondary antibody (ZhongShanJinQiao, BeiJing, China). After washing with PBS, ER-tracker was added and incubated at room temperature for 20 min. Following the washing of ER-tracker by PBS, DAPI was added. Cells were observed with a fluorescence microscope, and a fluorescence image was obtained.

### Flow cytometry

After digesting with ethylenediaminetetraacetic acid-free trypsin, the cells were collected by centrifugation at 300 g for 5 min at 4 °C. The cells were washed twice and centrifuged at 300 g with pre-cooled PBS, at 4 °C for 5 min. The cells were collected, and 100 μl of 1 × binding buffer was added to resuspend the cells. Five microliters of Annexin V-FITC and 5 μl of PI Staining Solution were gently mixed with cells at room temperature for 10 min in the darkness. Four hundred microliters of 1 × binding buffer was added to cells and mixed within 1 h. The compound was detected by flow cytometer Accuri C6 (Laboratories, Hercules, CA, USA).

### Cytoplasmic calcium concentration detection

Fluo-4AM dye (10 mM) was added to the cell culture medium to a final concentration of 10 μM. Cells were incubated at 37 °C, 5% CO_2_, for 1 h with Fluo-4AM dye. Then, it was replaced with fresh medium and incubated for 20 min. The cells were washed with PBS twice within 30 min and observed under a fluorescence microscope.

### Immunohistochemistry

The expressions of BAP31 (Cell Signaling Technology, MA, USA) proteins were studied by IHC in the CRC samples and pericarcinous tissues. Paraffin-embedded biopsies were included in tissue microarrays as previously described^49^. The sections were incubated at room temperature for 2 h with the indicated antibodies, and the immunostaining was performed using the ChemMate DAKO EnVision Detection Peroxidase/DAB kit (DAKO Diagnóstics, Barcelona, Spain).

### Western blot analysis

Western blot analysis was performed as described previously^50,51^. In brief, the samples were loaded on to 10% sodium dodecyl sulphate-polyacrylamide gels and transferred to polyvinylidene difluoride membranes (Millipore Corp, Bedford, MA, USA). Primary antibody dilutions were as follows: BAP31 (1:1000), GRP78/BIP (1:1000), PERK (1:2000), BAX (1:1000), and GAPDH (1:2000) (Proteintech, Rosemont, USA), and HRP-conjugated rabbit or mouse secondary antibody (1:10,000). Signal intensity was detected using a Chemiluminescence Kit (Thermo Fisher Scientific Fremont, CA, USA) on ChemiDoc™ Touch Imaging System (Bio-Rad, Hercules, California, USA, USA) and quantitated using ImageJ 1.42 software (National Institutes of Health, San Leandro, California, USA).

### In vivo experiments

Four-week-old male BALB/c-nu mice were obtained from Chengdu Dashuo Laboratory Animal Co., Ltd. (Chengdu, China) and were housed in a specific pathogen-free environment. Twenty 5-week-old BALB/c-nu male mice were randomly assigned into four groups: pcDNA-NC control group, pcDNA-miR-451a group, psilencer-Scr control group, and psilencer-BAP31 group, each group with five mice. This experiment was in accordance with the National Institutes of Health Guide for the Care and Use of Laboratory Animals and was approved by the Ethics Committee of West China Hospital (No. K2016040). CRC cell line, HCT116, was collected with 0.24% trypsin-ethylenediaminetetraacetic acid solution, washed, and suspended in the serum-free medium. HCT116 cells (8 × 10^6^) were subcutaneously injected into nude mice. Tumor volume was assessed by measuring the length (*L*) and width (*W*) with a caliper (tumor volume, *V* = 0.5 × *L* × *W*^2^). The body weights and tumor volumes were determined every 3 days. PcDNA-NC, pcDNA-miR451a, psilence-Scr, and psilence-BAP31 were intratumorally or peritumorally injected once a week for 3 weeks. The mice were sacrificed when administrated with vectors for 21 days. The tumors were collected and weighed, respectively. Total RNA and protein were extracted from tumors. Real-time PCR and western blot were carried out to detect the relative expression of miR-451a and BAP31 in tumors from each group.

### Statistical analysis

The data were expressed as the mean ± standard deviation. All histograms were carried out using GraphPad Prism 5.0 software. The Statistical Package for Social Sciences version 13.0 (SPSS Inc., Chicago, Illinois, USA) was used for standard statistical analysis by one-way analysis of variance. The relative gene expressions were analyzed with Livak method^52^. Pearson’s Chi-square test was used to compare the pathological data from the clinic^53^. The statistical significance was set at *P* < 0.05.
